# Increased Capitation Grants to Civil Hospitals

**Published:** 1918-02-23

**Authors:** 


### INCREASED CAPITATION GRANTS TO CIVIL HOSPITALS.

The following Army Council Instruction, dated February 10, 1918, has been issued:—

**144. Capitation Grant for Maintenance of Sick or Wounded Soldiers in Civil Established Hospitals.**

1. With reference to W.O. letter, Gen. No. 16/3042 (A.M.D. 2) of September 30, 1914, authorising the payment of a capitation rate not exceeding 4s. a day for soldier patients under treatment in civil established hospitals, it has been decided that the rates now paid may be increased to the extent of 9d., as from October 1, 1917, and that, in future, the maximum grant will be 4s. 9d.

2. An increase in existing rates may, therefore, be approved by G.Os.C.in-C. on application being made by the hospital concerned, and future applications will be dealt with as at present, except that grants may be authorised up to 4s. 9d. instead of to 4s. only. Increases within the maximum of 4s. 9d., other than that of 9d. addition to existing grants, should be granted only on the production of evidence that the resources and expenditure of the hospital necessitate the increase, and it should be a condition of such increase that any information required by the W.O. with regard to food consumption will be furnished.

3. From October 1, 1917, a grant of 6d. per diem for each unoccupied bed held at the disposal of the military authorities by their desire, may also be authorised.

4. (i) No other assistance, whether by way of grant or in kind, will be given to these hospitals without W.O. authority. In the event of special consideration being asked for, applications for a special donation in addition to the maximum capitation grant will be considered where it can be shown that, owing to exceptional circumstances, the maximum grant is insufficient for the expenses attendant upon the treatment of military patients.

(ii) Such exceptional circumstances will be limited to the following: (*a*) Hospitals with fully-equipped pathological departments; (*b*) Hospitals with special massage facilities or *x*-ray apparatus, enabling them to deal with certain classes of patients; (*c*) Hospitals which pay a full-time salary in respect of resident medical officers; (*d*) Hospitals which, since the beginning of the war have incurred expenditure on buildings for military patients or on lighting or upkeep of an exceptional character.

(iii) Applications for a special donation will be submitted through Command Headquarters with a full statement of the grounds on which the application is based.

(iv) In considering each case, the Army Council will require detailed information as to accounts, expenditure, food consumption and methods of administration in the hospital in question. 53/Gen. No./8548 (F.2).

By Command of the Army Council,

R. W. Brade.

[Copies for G.Os.C.-in-C. and G.Os.C. at Home (and for distribution to all military and auxiliary hospitals in established civil institutions); Command Paymasters; Local Auditors.]

